# Evaluation of predictive role of carcinoembryonic antigen and salivary mRNA biomarkers in gastric cancer detection

**DOI:** 10.1097/MD.0000000000020419

**Published:** 2020-05-29

**Authors:** Fei Xu, Meiquan Jiang

**Affiliations:** a Department of Digestive Medicine; b Department of General Surgery, PKUCare Luzhong Hospital, Zibo, Shandong, China.

**Keywords:** blood, carcinoma, carcinoembryonic antigen, liquid biopsy, messenger RNA, saliva, stomach

## Abstract

We explored the potential of combining carcinoembryonic antigen (CEA) and salivary mRNAs for gastric cancer (GC) detection.

This study included 2 phases of study: a biomarker discovery phase and an independent validation phase. In the discovery phase, we measured CEA levels in blood samples and expression level of messenger RNAs (SPINK7, PPL, SEMA4B, SMAD4) in saliva samples of 140 GC patients and 140 healthy controls. We evaluated the clinical performance of each biomarker and developed a predictive model using machine-learning algorithm to differentiate GC patients and healthy controls.

Our biomarker panel successfully discriminated GC patients from healthy controls with both high sensitivity (0.94) and high specificity (0.91). We next applied our biomarker panel in the independent validation phase, in which we recruited a new patient cohort of 60 GC patients and 60 healthy controls. Using our biomarker panel, the GC patients were discriminated from healthy controls in the validation phase, with sensitivity of 0.92 and specificity of 0.87.

A combination of blood CEA and salivary messenger RNA could be a promising approach to detect GC.

## 1 Introduction

Gastric cancer (GC) is estimated to cause the third most deaths amongst all types of cancer in the world, trailing only lung cancer and colorectal cancer.^[1]^ In Eastern Asia, the occurrence of GC is the highest for both males and females (32.1% and 12.2%, respectively).^[1]^ In China alone, GC leads to an incidence rate as high as 29.9%, and contributes to almost 50% of the world's GC deaths.^[2]^ Infection of *Helicobacter pylori*, a bacterium living in the human stomach, consumption of salt-preserved foods, low intake of fruit, consumption of alcohol, and active tobacco smoking^[1,3]^ are top risk factors for GC. Till now, majority of the GC patients are not identified until their late stages. In China, the 5-year survival rate of GC is only 14% for stage IV patients, compared to 78% for stage I patients.^[2]^

Carcinoembryonic antigen (CEA) has been commonly used as a non-invasive cancer biomarker in multiple clinical settings. CEA can detect the recurrence of colorectal cancer with high evidence of clinical utility, and also help determine the response or progression in metastatic breast cancer.^[4]^ CEA test is considered non-invasive as it can be detected in blood samples. However, CEA alone does not have enough discriminatory power for cancer detection due to its high false positive rate, and many patients with benign gastric diseases could receive positive results.^[5]^

Messenger RNAs (mRNAs) and microRNAs (miRNA) in saliva have been recently identified as promising biomarkers for GC detection, mainly because of their non-invasive nature. The principle of using salivary RNA for GC detection has been reported.^[6]^ Basically, during the development of GC, salivary gland was stimulated by mediators such as nerve growth factor released from remote tumors. This led to significant changes of salivary RNA profiles, which could be used for GC detection. Li et al found that a panel of 3 mRNAs (SPINK7, PPL, and SEMA4B) and 2 microRNAs (MIR140-5p and MIR301a) can discriminate GC patients from healthy controls with credible clinical performance.^[7]^ Using a multiple logistic model, Li et al maximized the sensitivity and specificity to be 75% and 83%. One limitation of the study of Li et al was that only Korean population was included in the patient cohort and thus, whether its findings could be transferred into other races such as Chinese population remains unknown. In addition, it is worth mentioning that salivary mRNA has been investigated for other types of cancer, including oral cancer,^[8–11]^ ovarian cancer,^[12]^ breast cancer,^[13]^ lung cancer,^[14]^ and pancreatic cancer.^[15]^

So far, most of the liquid biopsy studies on detecting GC were conducted by using either blood biomarkers such as CEA or salivary biomarkers such as mRNA biomarkers. This study aimed at exploring the potential of combining blood biomarkers and salivary biomarkers to detect GC. To this end, we designed our study with 2 phases: a discovery phase to find and evaluate biomarkers, and an independent validation phase to confirm the applicability of the selected biomarkers. In an initial discovery phase, blood samples and saliva samples were collected from 140 GC patients and 140 healthy controls. The enzyme immunoassays were applied to measure CEA level in blood samples, and RT-qPCR was used to measure the expression levels of 4 candidate mRNA biomarkers in saliva samples, namely SPINK7, PPL, SEMA4B, and SMAD4. We adopted this panel of RNA biomarkers in a Chinese population to explore its clinical application in different populations and also the combined use with CEA analysis to improve GC detection. Using a machine-learning algorithm that was trained with the measurement data, we successfully discriminated GC patients from healthy controls: the sensitivity reaching 0.94 and the specificity reaching 0.91. Next, we conducted an independent clinical validation phase by recruiting a separate cohort of participants with 60 GC patients and 60 healthy controls. In this phase of study, CEA level in blood and mRNA level of PPL in saliva of were measured similarly as that in the discovery phase. By applying our machine-learning algorithm that was previously trained, GC patients and healthy controls were again differentiated with high sensitivity of 0.92 (55/60) and high specificity of 0.87 (52/60).

## 2 Materials and methods

### 2.1 Patients and specimens

The institutional review board at PKUCare Luzhong Hospital approved this study (2015-KY-008). To perform a clinical trial in the biomarker discovery phase, we recruited 140 GC patients from May 12, 2017, to February 14, 2018, from PKUCare Luzhong Hospital (Zibo, Shandong, China). Simultaneously, 140 non-GC people (healthy controls) were randomly selected as blood and saliva donors for the study. We obtain informed consent forms from all research participants. Similar as the patient recruitment in the discovery phase, 60 GC patients, and 60 healthy controls were recruited in the independent validation phase between February 26, 2018, and July 10, 2018, from PKUCare Luzhong Hospital. Table 1 presented the details on characteristics of patients that were recruited in both the biomarker discovery phase and the validation phase.

**Table 1:**
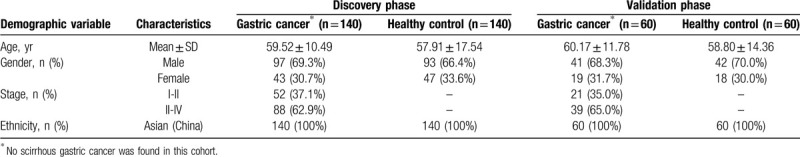
Demographic information of all subjects used in this study.

### 2.2 Collection of blood samples and measurement of CEA level

The same procedures as listed below were used to collect blood samples of participants in both the biomarker discovery phase and validation phase. In brief, a total of 2 mL peripheral blood samples were obtained between 08:00 and 09:00 am from GC patients and healthy controls. Anti-coagulant was not used when collecting blood samples for measuring CEA concentrations. Sera were then separated from blood samples for each sample within 30 minutes after collection, followed by using a commercial Luminex xMAP assay (Luminex, Austin, TX) to detect CEA. We performed the multiplexed analyses according to the assay protocol and analyzed the intensities of fluorescence by using the Bio-Plex suspension array system (Bio-Rad Laboratories, Hercules, CA). The final concentration of CEA was calculated by referencing to a standard curve using the system software.

### 2.3 Collection of saliva samples and measurement of mRNA levels

The same procedures as listed below were used to collect saliva samples of participants in both the biomarker discovery phase and validation phase. In brief, each participant was instructed to avoid drinking, smoking, eating, and oral hygiene procedures for 1 hour or longer before sample collection. We then collected a total of 2 mL saliva samples for each participant and kept them on ice. To measure the mRNA level in saliva samples, the samples were first centrifuged at 13,000 g for 10 minutes at 4°C. We then removed the supernatant and treated it with RNase inhibitor (Superase-In, Ambion Inc., Austin, TX) to prevent RNA degradation. The saliva samples were stored at −80°C before proceeding to RNA extraction and measurement. To extract and measure mRNA level in saliva, we followed a protocol that was previously reported.^[14]^ In general, RNA was extracted from 330 μL saliva supernatant by using RNeasy Protect Saliva Mini Kit (Qiagen, Germany), followed by linear amplification by using TURBO DNase treatment. We then applied reverse transcription quantitative real-time PCR (qRT-PCR) analysis on selected genes. The qRT-PCR amplification was conducted in 20 μL reactions of Roche LightCycler 480 (Roche, Switzerland) by following the manufacturer's protocols. Table 2 listed the primers used in qRT-PCR. To quantify the relative mRNA level, we used the ΔCt method as previously reported.^[16]^ In short, we chose GAPDH as internal control. The relative expression level of selected gene was calculated as 2 ^ (- ΔCt) multiplying 1000, where ΔCt represented normalized Ct values as Ct of selected gene – Ct of GAPDH.

**Table 2:**
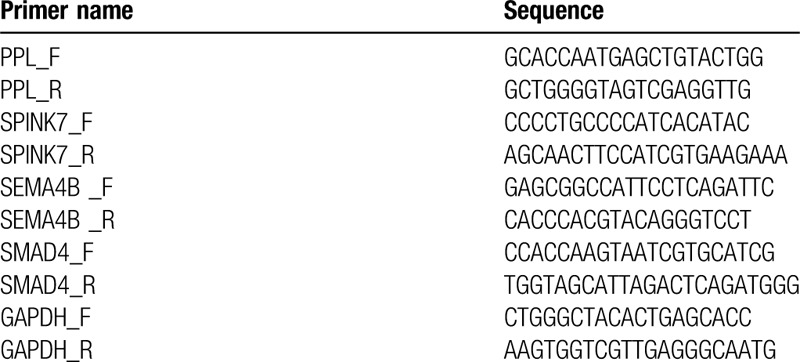
Primers used in this study.

### 2.4 Statistical methods and machine learning analysis

All statistical analyses were performed using GraphPad Prism (GraphPad Software, La Jolla, CA). A 2-sided *P*-value <.05 was selected as the cut-off for statistical significance when comparing CEA level or mRNA level between patient group and control group. The discriminatory power of each biomarker was determined by receiver operating characteristic (ROC) analysis and calculating the area under the curve (AUC). A biomarker with AUC value larger than 0.70 was treated as discriminatory. Then, we used the discriminatory biomarkers to develop predictive models by using machine learning analysis. Least absolute shrinkage and selection operator (LASSO) algorithm was selected as our classifier for data collected in the biomarker discovery phase, LASSO is a well-known, penalized method suitable for classifying high-dimensional data and avoiding overfitting.^[17,18]^ We conducted LASSO analysis using python scikit-learn package. To further determine if our classifier overfitted the result, we performed 10-fold cross-validations, i.e., splitting the training datasets randomly into 10 parts, using 9 parts for training and the rest 1 part for validation, and repeating the entire process for 10 times. After the classifier was trained, we then used the classifier to predict the occurrence of GC with the data collected in the validation phase.

## 3 Results

### 3.1 Overview of study design

**Figure 1:**
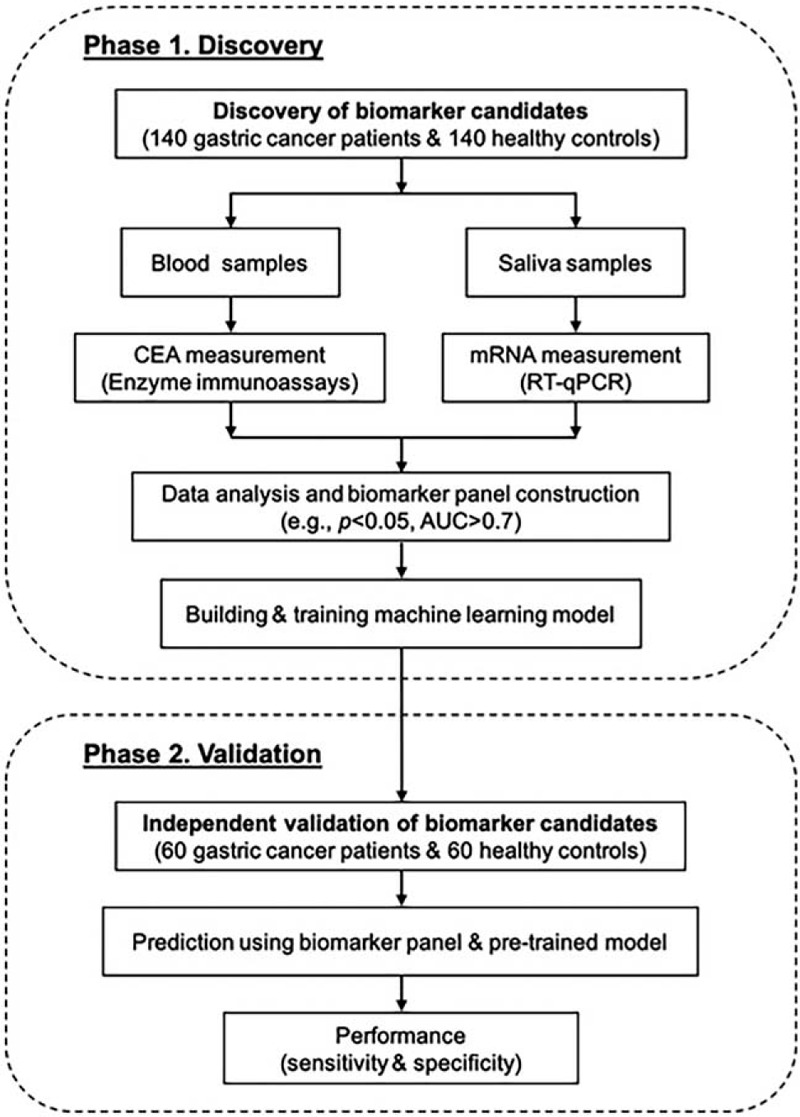
Flowchart of the study design for development of a novel gastric cancer biomarker panel.

We designed our study with 2 phases (Fig. 1), that is, a biomarker discovery phase (phase I) and a clinical validation phase (phase II). The biomarker discovery phase aimed at identifying and evaluating the classification performance of candidate biomarkers in separating GC patients from healthy controls. A total of 140 GC patients and 140 healthy controls were recruited in the discovery phase. For each participant, we measured the CEA level in blood and mRNA levels of 4 candidate biomarker genes in saliva. We next developed a predictive model that used a few selected biomarkers to determine whether or not a sample was from GC patient. Next, we designed the validation phase, which aimed at evaluating the clinical performance of the biomarker panel identified in the discovery phase. Totally 60 GC patients and 60 healthy controls were recruited as an independent patient cohort in the validation phase, followed by blind testing of their biomarker level in blood and saliva. Using the predictive model developed in the discovery phase, we then made predictions on if a sample of the validation phase was from a GC patient. Finally, we compared our predictions with pathological classification to quantitatively calculate sensitivity and specificity as the measure of clinical performance of our biomarker panel.

### 3.2 Identify biomarkers from the patient cohort of the discovery phase

**Figure 2:**
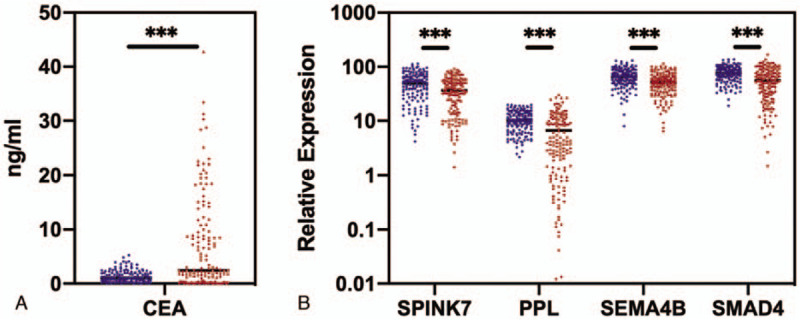
Comparison of carcinoembryonic antigen levels in blood samples (A) and messenger RNA expression levels of candiate genes in saliva samples (B) between healthy control group (blue) and gastric cancer patient group (red) in the discovery phase. The healthy controls were chosen as biological references. The healthy control group included 140 healthy controls, while the gastric cancer patient group included 140 patients. ^∗∗∗^ indicates *P* < .001.

We measured 2 types of biomarkers from the samples collected in the discovery phase: CEA level in blood, and mRNA level of SPINK7, PPL, SEMA4B, and SMAD4 in saliva. These 4 RNAs were chosen as candidate biomarkers because they have been previously reported^[7]^ to be effective in discriminating GC patients from healthy controls in Korean population. However, whether or not these biomarkers are effective in detecting GC in Chinese population remains unknown. Therefore, we aimed to choose these 4 RNAs as candidate biomarkers and evaluated their clinical performance in differentiating GC patients from healthy controls in Chinese population. We then compared the biomarker levels between GC patients (n = 140) and healthy controls (n = 140). We found that the CEA level was significantly elevated (*P* < .001) in the GC patients (mean = 6.91 ng/mL, Fig. 2) compared to that of the healthy controls (mean = 1.35 ng/mL). The expression level of each individual mRNA biomarkers in saliva was significantly down regulated in GC patients (*P* < .001) compared to that of the healthy controls, with PPL demonstrating highest decreases of expression (1.55-fold) in GC patients (*P* < .001). This indicated that CEA level in blood as well as mRNA expression in saliva could be potentially used to discriminate GC patients from healthy controls.

### 3.3 Biomarker evaluation and predictive model development

**Figure 3:**
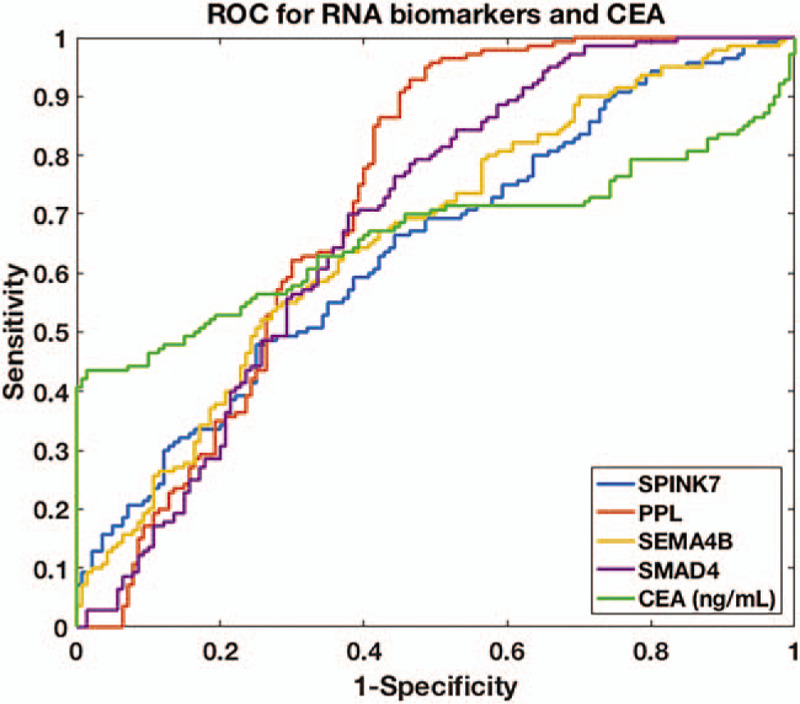
Receiver operating characteristic curves of single messenger RNA biomarkers (SPINK7, PPL, SEMA4B and SMAD4) in saliva and carcinoembryonic antigen in blood in the discovery phase.

To further evaluate a biomarker's performance in discriminating GC patients from healthy controls, we applied receiver operating characteristic curve for each biomarker together with the calculation of the AUC value (Fig. 3). We set our cut-off value of AUC as 0.70, that is, a biomarker with AUC value over 0.70 was considered to demonstrate decent performance for discriminating GC patients from healthy controls. Among the 5 biomarkers evaluated, PPL had the highest AUC value as 0.72 while the rest biomarkers had AUC values lower than 0.70, indicating that solitary use of biomarkers led to unsatisfactory clinical performance for discriminating GC patients and healthy controls.

**Figure 4:**
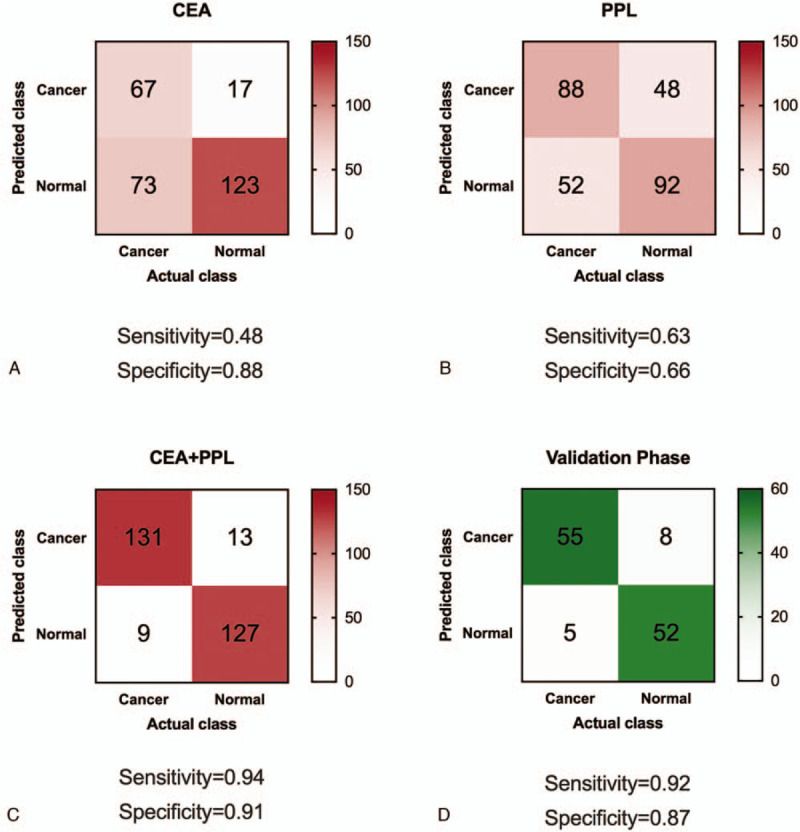
Confusion matrix of different panels of biomarkers in the discovery phase (A, B, C) and the validation phase (D).

Next, we developed a machine-learning-based model to predict occurrence of GC. We chose 2 biomarkers, that is, PPL level in saliva and CEA level in blood, as the model input. The rationale of including CEA level in blood was due to its wide application in clinical blood tests as well as its board line value of AUC (AUC = 0.66). We applied LASSO algorithm as our classifier and used the paired data of biomarker level and GC occurrence of the 140 participants in the discovery phase to train the model. When using solitary biomarker (either PPL or CEA), we could only achieve fairly well predictions with sensitivity reaching 0.48 to 0.63 and specificity reaching 0.66 to 0.88 (Fig. 4). However, when combining both PPL and CEA in our model, both sensitivity and specificity were dramatically improved: sensitivity was increased to 0.94 and specificity was increased to 0.91. This suggested that combined use of blood biomarker and saliva biomarker could effectively discriminate GC patients from healthy controls.

### 3.4 Biomarker validation in an independent patient cohort

In order to evaluate the clinical performance of the combined use of blood biomarker (i.e., CEA level) and saliva biomarker (i.e., PPL expression level), we recruited an independent patient cohort with 60 GC patients and 60 healthy controls in the validation phase. The samples collected in this validation phase were blinded. For each participant, we measured the CEA level in blood and PPL expression level in saliva, and next applied the LASSO algorithm developed in the discovery phase to make predictions on GC occurrence. In sum, 52 of 60 healthy controls as well as 55 of 60 GC patients were correctly predicted, leading to sensitivity of 0.92 and specificity of 0.87 (Fig. 4D). The high sensitivity and high specificity confirmed the applicability of the combined use of blood biomarker and saliva biomarker as a novel non-invasive method for GC detection.

## 4 Discussion

In this study, we proved that combining multiple biomarkers from blood and saliva could improve the sensitivity and specificity in detection of GC. Our study complemented past and current endeavors on developing liquid biopsy approaches for GC detection. It is worth noticing that using multi-analytes for cancer detection is becoming an emerging field of liquid biopsy. Recently, CancerSEEK^[19]^ analyzed multiple mutations of ctDNA in blood as well as multiple protein biomarkers and successfully detected a wide range of cancers (e.g., liver cancer and ovary cancer) with both high sensitivity and high specificity. We noticed that the sensitivity of CancerSEEK in GC detection was about 70%, while our biomarker panel could achieve sensitivity as high as 92%. This suggested that combined biomarker analysis had large room for further improvement and calls for more research efforts.

We would also like to highlight the key of this study as well as limitations. Using machine learning to develop a predictive model for GC prediction was one of the key elements in this study. Nowadays, machine learning has been widely applied in various fields of biomarker analysis, including ctDNA,^[20]^ ctRNA,^[21]^ proteomics,^[22,23]^ and metabolomics.^[24]^ Similar to previous reports, we also found that using machine learning for model development dramatically increased the classification performance of the biomarker panel. We also want to point out that to avoid potential overfitting issue of machine learning-based models, we deliberately included an independent validation phase to test if the clinical performance of our biomarker panel remained consist between the biomarker discovery phase and the validation phase. This step is crucial as the model may be skewed with high accuracy in the discovery phase but performs poorly when being deployed for real-world testing. In this study, we found the sensitivity and specificity between the biomarker discovery phase and the validation phase was similar, which supported the conclusion that our biomarker panel could be potentially adopted for clinical applications. Additionally, our method could be further integrated with other novel methods to screen GC, such as a screening method for GC by oral microbiome detection that was recently developed with high sensitivity.^[25]^

We also noticed a few limitations of this study. For example, the participants of this study were recruited from China. Therefore, it remained partially unknown how the biomarker panel discovered in this study would perform in other races in detection of GC. Also, the aim of this study focused on discovering and validating a biomarker panel for GC. Investigating the molecular mechanisms of the biomarkers as well as holistically exploring the landscape of all possible GC-related biomarkers is beyond the scope of this study. That being said, certain biomarkers that were previously reported to be associated with GC development were not tested in this study. For example, APC,^[26,27]^ BMPR1A,^[28,29]^ CDH1,^[26,30]^ CSTB,^[26,31]^ EPCAM,^[27,29]^ MLH1,^[28,29]^ MSH2,^[26,27]^ MSH6,^[27,32]^ STK11,^[27,31]^ TP53,^[30,31]^ and TPI1^[27,33]^ could be promising candidate biomarkers, because these genes are relevant to GC occurrence. To this end, we are collaborating with researchers in U.S. and Europe to conduct a large-scale, multi-center, multi-marker study to systematically evaluate the GC biomarkers. Finally, our study did not cover all types of gastric carcinoma. Several rare types of gastric carcinoma such as scirrhous GC were not found in our cohort of study. CEA and other tumor markers are sometimes not increased in patients with scirrhous GC. Therefore, whether or not our biomarker panel can be extended to rare GCs requires further validation.

To conclude, we proved the concept of combining biomarkers from blood samples and saliva samples could improve the accuracy of non-invasive detection of GC. The biomarker panel discovered in this study presents a promising non-invasive approach for GC detection.

## Author contributions

**Conceptualization:** Fei Xu, Meiquan Jiang

**Data curation:** Fei Xu

**Investigation & Supervision:** Meiquan Jiang

**Writing – original draft:** Fei Xu

**Writing – review & editing:** Meiquan Jiang
